# Thymoma and thymic carcinoma associated with multilocular thymic cyst: a clinicopathologic analysis of 18 cases

**DOI:** 10.1186/s13000-018-0719-7

**Published:** 2018-06-26

**Authors:** Xuxia Shen, Yan Jin, Lei Shen, Yihua Sun, Haiquan Chen, Yuan Li

**Affiliations:** 1 Department of Pathology, Fudan University Shanghai Cancer Center, 270 Dong-An Road, Shanghai, 200032 China; 2 Department of Thoracic Surgery, Fudan University Shanghai Cancer Center, Shanghai, China; 3 Department of Oncology, Shanghai Medical College, Fudan University, Shanghai, China

**Keywords:** Multilocular thymic cyst, Thymoma, Thymic carcinoma

## Abstract

**Background:**

Multilocular thymic cysts (MTCs) associated with thymomas or thymic carcinomas (TCs) are rare and may be misdiagnosed as other benign cystic lesions.

**Methods:**

We retrospectively analysed 18 cases of thymomas or TCs associated with MTCs, which were retrieved from 309 consecutive patients with thymomas or TCs in the Chinese population, emphasizing clinicopathologic characteristics, immunophenotypes and the prognostic impact.

**Results:**

A total of 14 tumours were described as cystic or solid-cystic masses, and the other 4 tumours were described as solid masses. Histologically, 2 atypical type A, 2 type AB, 1 type B1, 8 type B2, 1 type B3, 1 microscopic thymoma (type A), 2 squamous cell carcinomas (SCCs) and 1 lymphoepithelioma-like carcinoma (LELC) were classified. Prominent multilocular cystic areas with chronic inflammation were observed. The follow-up ranged from 2 to 79 months. Sixteen patients survived without any evidence of recurrence after complete resection.

**Conclusions:**

Our study suggests that thymomas or TCs associated with MTCs are rare in the Chinese population and have a better clinical behaviour than thymomas or TCs without MTCs. Our data also expand the histologic spectrum of thymomas or TCs accompanied by MTC. To our knowledge, this is the first report of atypical type A thymoma and LELC associated with MTCs.

## Background

Thymomas and TCs are considered as a series of thymic epithelial tumours of low malignant potential to malignant, which encompass a group of different morphologic neoplasms. Most TCs can be classified as SCCs, but several uncommonly histological variants exist, including LELC, basaloid carcinoma, clear cell carcinoma, neuroendocrine carcinoma, mucoepidermoid carcinoma and sarcomatoid carcinoma, etc. [1] The 2015 World Health Organization (WHO) classification subdivides thymomas into type A, AB, B1, B2, B3 and rare other thymomas, and defines atypical type A thymoma as type A thymoma variant [1]. The major criteria of the new concept of atypical A thymoma are increased mitotic activity (4 or more per 10 high power field) and coagulative tumour necrosis. Other criteria are difficult to quantify or could not be agreed upon, including hypercellularity, enlarged hyperchromatic nuclei, large nucleoli, increased Ki-67 index, and the extent of atypical areas [1, 2].

MTCs are uncommon lesions and considered to be acquired lesions of the thymic gland secondary to various inflammatory diseases. MTCs associated with thymomas or TCs are relatively rare and have sporadically been described. To our knowledge, no more than 50 cases have been reported in the English literature [3–13]. MTCs with thymomas or TCs may be clinically misdiagnosed as other benign cysts of the mediastinum. In addition, the overall rarity of the epithelial thymic tumours associated with MTCs impedes the establishment of unified and reliable conclusion with regard to therapy and prognosis. Herein, we present a retrospective study on the clinicopathologic characteristics of 18 cases of epithelial thymic tumours associated with MTCs. Additionally, we attempt to explore the formation mechanism of MTCs through immunohistochemistry staining and review of the literatures. As far as we know, this is the first report to investigate MTCs associated with atypical type A and LELC.

## Methods

A total of 309 in-house and consultation cases of surgically excised thymomas and TCs performed between 2008 and 2016 were retrieved from the pathology files of Shanghai Cancer Center, China. Our data were approved by the ethics committee of Fudan University Shanghai Cancer Center. Four to ten haematoxylin and eosin-stained (HE) slides from each case were reviewed by three thoracic pathologists. Eighteen cases (6 consultation patients and 12 in-house patients) showed the features described above. The metastasis to the thymus was excluded. We retrospectively studied the clinicopathologic features of these 18 cases, including the clinical information, radiographic images, histologic and immunohistochemical features. All clinical data were obtained from the clinical charts or directly from the referring clinician. Representative paraffin blocks were selected for immunohistochemistry. Immunostaining was performed on 4 mm thick formalin-fixed paraffin-embedded (FFPE) sections. Tissue sections were deparaffinised and pre-treated with 1 mmol/L EDTA and heat-mediated antigen retrieval solution in a microwave oven. Further steps were done at room temperature in a hydrated chamber. Slides were preincubated in 20% normal goat serum, and then incubated with antibodies against pan-cytokeratin (AE1/AE3; 1:100; Dako, Carpinteria, CA), CK5/6(1:50, Dako, Carpinteria, CA), p63 (1:50; Dako, Carpinteria, CA), TdT (1:100, Dako, Carpinteria, CA), CD20(1:50, Dako, Carpinteria, CA), CD117(1:200; Dako, Carpinteria, CA) and CD5 (1:20; Thermo Fisher Scientific, Fremont, CA). The slides were then washed in Tris-HCl and detected with horseradish peroxidase-conjugated anti-rabbit EnVision+ kit (Dako). All slides were counterstained with haematoxylin. Adequate positive and negative controls were performed in all antibodies tested. The Epstein-Barr virus (EBV) hybridization in situ was performed on FFPE specimens.

## Results

### Clinical features

Table 1 summarizes the clinical features of all 18 cases. Patients were composed of 10 males and 8 females, their ages ranging from 24 to 67 years, with an average age of 45 years (median, 43.5 years). All 18 patients’ clinical and follow-up data were obtained. Of 18 patients, 11 presented with chest pain, discomfort, dyspnoea, cough, and/or myasthenia, and no information regarding presenting signs or symptoms was available for the remaining patients. Of the 7 asymptomatic patients, 6 were incidental findings on computed tomography (CT) scans and 1 on PET-CT scan. All 18 patients underwent complete surgical resection, and 10 of these patients underwent radiotherapy, and 1 patient underwent combined adjuvant chemotherapy. According to the WHO TNM classification [1], 11 cases were stage I, 6 were stage II and 1 was stage IV. The follow-up ranged from 2 to 79 months (average 26.7 months; median, 17.5 months). Sixteen patients were alive without any evidence of thymoma or TC recurrence after complete resection. One patient recurred in lymph node metastasis of right supraclavicular lymph nodes 7 months after surgery, and was alive with disease at 12 months. A new neoplasm of chondrosarcoma occurred in one patient 6 months after surgery, and then was lost to follow-up.

**Table 1:** The clinicopathologic features of the 18 patients with thymomas and TCs associated with MTCs

Case	Sex	Symptom	CT scan (with MTC or not)	Tumor size (cm)	Gross description	Tumor type	Stage	Follow up
1	M	asymptomatic	Yes	5	solid-cystic	SCC	I	AW at 23 mo
2	F	chest pain and discomfort	No	7.5	solid-cystic	atypical A	I	AW at 21 mo
3	M	asymptomatic	No	15	solid-cystic	B2	I	Chondrosarcoma occurred at 6 mo, then lost follow-up
4	M	cough	No	10	solid-cystic	atypical A	I	AW at 17 mo
5	M	asymptomatic	No	6.5	solid-cystic	B2	II	AW at 53 mo
6	F	cough and chest pain	Yes	8	solid-cystic	B3	II	AW at 20 mo
7	M	chest pain	No	9	solid-cystic	B2	I	AW at 79 mo
8	M	cough and chest pain	No	8.5	solid	AB	I	AW at 53 mo
9	F	asymptomatic	Yes	9	solid	B2	II	AW at 75 mo
10	F	chest pain	No	10.5	solid	B2	II	AW at 58 mo
11	F	asymptomatic	Yes	12	cystic	Microscopic thymoma (type A)	I	AW at 18 mo
12	M	cough and chest pain	No	7	solid-cystic	LELC	IV	AWD at 12 mo
13	M	chest pain	Yes	3.5	solid-cystic	B2	I	AW at 11 mo
14	F	myasthenia	Yes	8.5	solid-cystic	B2	II	AW at 11 mo
15	F	dyspnea and chest pain	No	3	solid	AB	I	AW at 14 mo
16	M	myasthenia	Yes	2.5	solid-cystic	B2	I	AW at 6 mo
17	F	asymptomatic	No	8	solid-cystic	B1	I	AW at 2 mo
18	M	asymptomatic	Yes	7.5	solid-cystic	SCC	II	AW at 2 mo

*F* female, *M* male, *AW* alive and well, *AWD* alive with disease, *mo* months

### CT findings

**Fig. 1:**
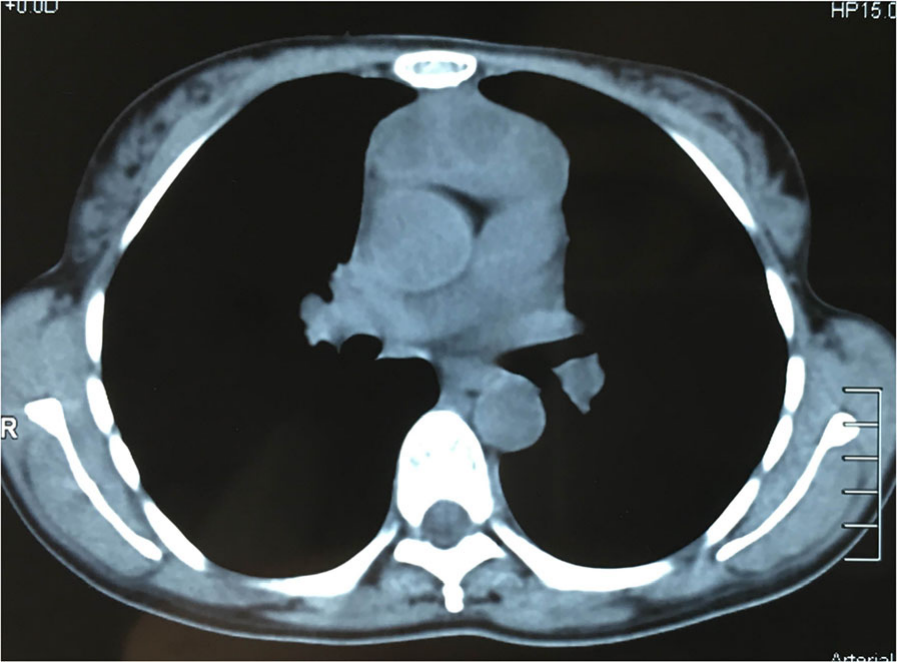
Computed tomographic findings: chest computed tomogram showed an anterior mediastinal mass composed of multilocular cyst

On CT or PET-CT, all cases showed anterior mediastinal masses inseparable from the thymus. In only 7 of 18 cases, MTCs were found adjacent to the thymoma or TC (Fig. 1), and one case showed a large unilocular cyst in the anterior mediastinum. In the other 10 cases, no cysts were examined around the tumours on the images, and 2 of the 10 cases, the tumour presented close neighbour to the pericardium.

### Pathological findings

**Fig. 2:**
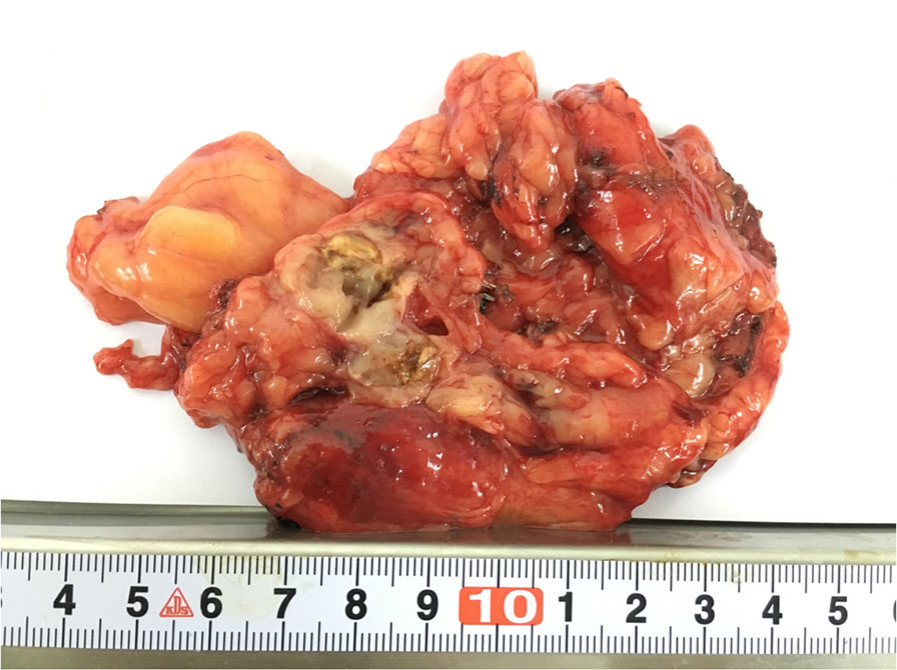
Gross picture of a type B2 thymoma associated with MTC

Grossly, the tumour size of the 18 cases ranged from 2.5 to 15.0 cm in dimension (average, 7.8 cm; median, 8.0 cm). Thirteen tumours showed solid-cystic masses. The cystic area was described as multilocular cystic spaces separated by a thin wall and the dimension of the individual cyst ranged from 0.5 to 2 cm. The multilocular cyst contained turbid, haemorrhagic or clear fluid. The cut surface of the solid area was white-yellow in color, and with areas of necrosis and haemorrhage (Fig. 2). In one case, the tumour was described as a pure multiple cysts and filled with clear fluid, and the greatest dimension of the largest cyst was 8 cm. The other 4 tumours were described solid and lobulated lesions which showed white-grey or tan-yellow cut surface.

**Fig. 3:**
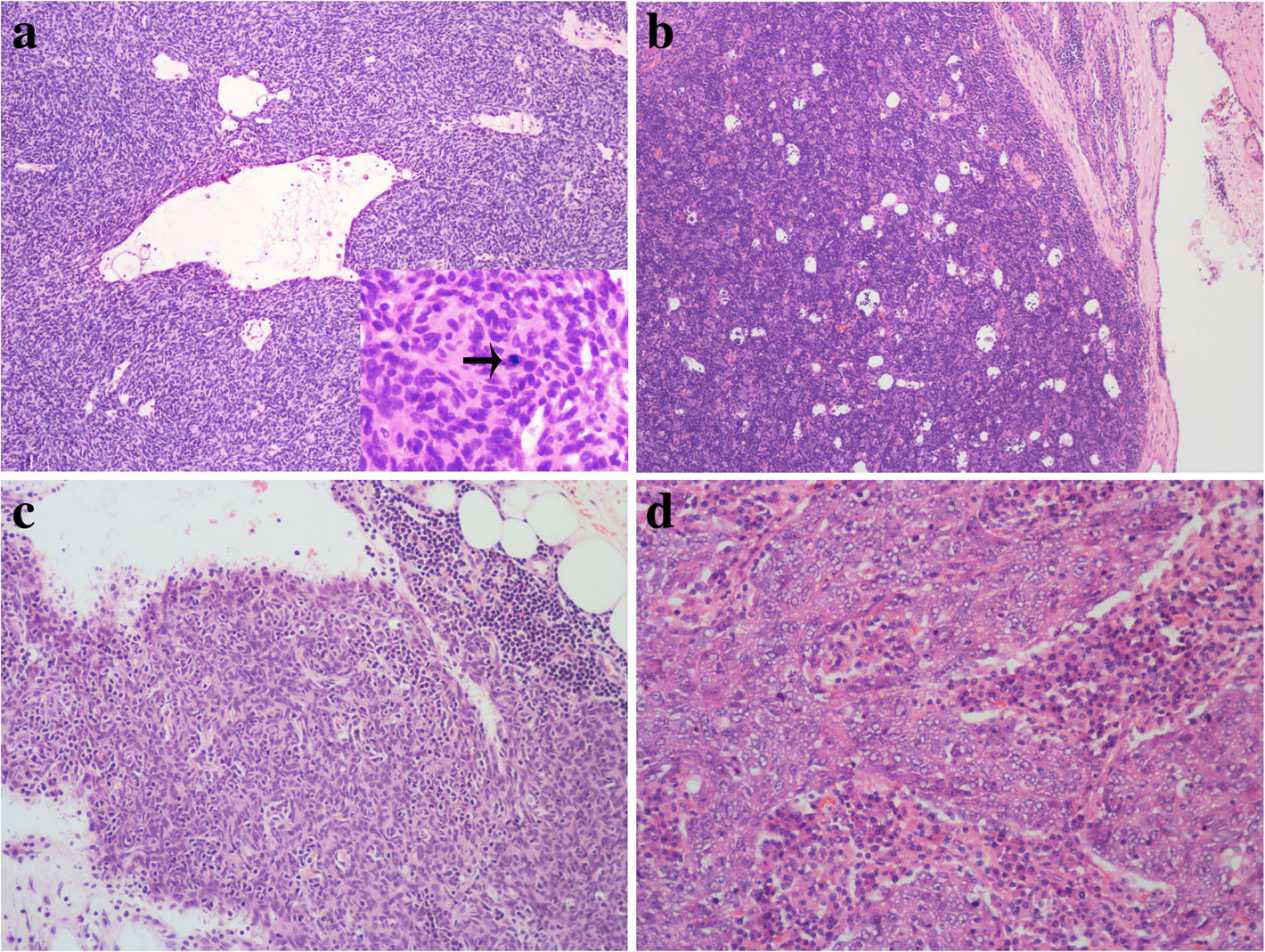
**a** An atypical type A thymoma showed the focal area of the PVS and mitoses (arrow). **b** Type B1 thymoma. **c** Microscopic thymoma (type A). **d** LELC

**Fig. 4:**
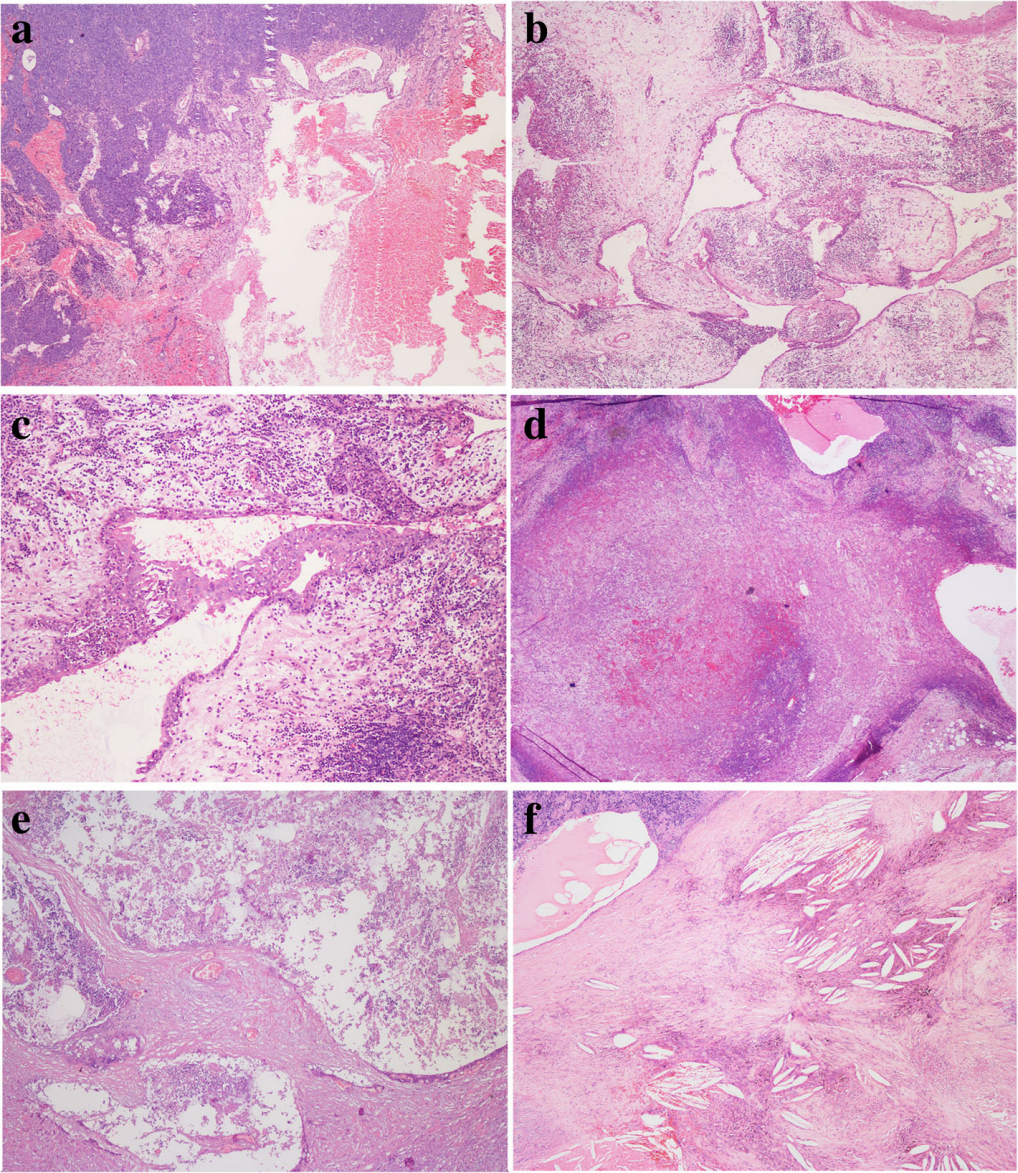
**a** Thymoma (left) adjacent to MTC (right), with haemorrhage. **b** A typical MTC showed remnant thymic tissue in the periphery of the cyst. **c** MTC lined by squamous epithelium with inflammatory cells infiltration. **d** Atypical type A thymoma was continuous with the cyst lining. **e** MTC displaying prominent necrosis. **f** MTC displaying cholesterol deposition

Applying the new edition of the histologic classification system of WHO [1], 2 atypical type A (Fig. 3a), 2 type AB, 1 type B1 (Fig. 3b), 8 type B2, 1 type B3, 1 microscopic thymoma (type A) (Fig. 3c), 2 SCC and 1 LELC (Fig. 3d) were observed in our study. The atypical type A thymomas consisted of spindle and oval-shaped tumour cells and which were characterized by occurrence of atypical areas with hypercellularity, enlarged hyperchromatic nuclei, and increased mitotic activity (5 and 4 per 10 high power field). Paucity or no immature thymocytes were found throughout the tumour. Focal area of perivascular space (PVS) was seen in one case. Syncytial growth of undifferentiated carcinoma cells in a lymphoplasmacytic stroma similar to undifferentiated carcinoma of the nasopharynx was observed in the LELC. The tumour cells had large vesicular nuclei with one or more prominent eosinophilic nucleoli. Lymphocytes were not only seen in the stroma, but were also admixed with the carcinoma cells intimately. The other 13 thymomas and 2 SCCs all showed typical histologic features of thymoma or carcinoma. In addition, the tumour components were small-sized and limited variably. All cases exhibited the typical features of MTCs accompanied by prominent chronic inflammation and fibrosis, including 2 type B2 and 2 type AB thymomas without cystic lesion grossly (Fig. 4a). The squamous, cuboidal or columnar epithelial lining of the cyst was partly preserved, and remnant thymic tissue was observed in the cyst wall and/or the periphery of the limited tumours nests (Fig. 4b and c). One microscopic (type A) and 2 atypical type A thymomas were observed continuous with the cyst lining (Fig. 4d). Other nonspecific changes, including haemorrhage, necrosis (Fig. 4e), edema and cholesterol deposition (Fig. 4f) were identified in the cyst wall.

**Fig. 5:**
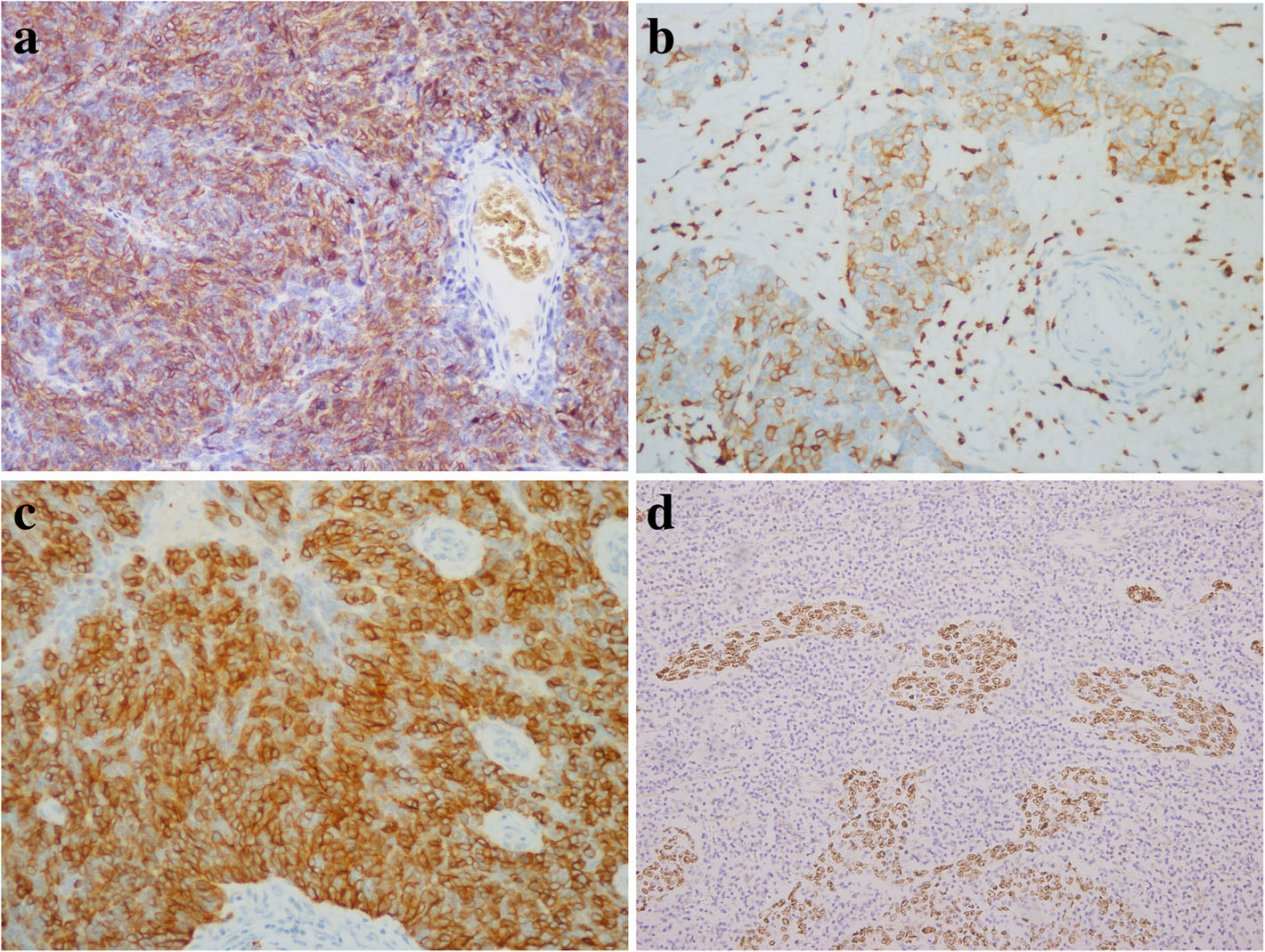
**a** Immunohistochemistry showed diffuse staining for CD20 in atypical A thymoma. **b** and **c** Immunohistochemistry showed strong and diffuse staining for CD5 and CD117 in SCC. **d** Representative image of EBV signals determined by hybridization in situ in LELC

All cases were performed immunohistochemical study. The neoplastic epithelia, the epithelial linings of the cystic wall and the Hassall corpuscles were strongly, focally or weakly positive for pan-cytokeratin (AE1/AE3), CK5/6 and p63, as expected. The neoplastic epithelia of atypical type A thymoma and microscopic thymoma (type A) showed diffuse reactivity for CD20 (Fig. 5a), and type AB thymoma showed focal reactivity for CD20. The tumour cells of LELC and SCC showed positivity for CD5 and CD117 (Fig. 5b and c). The immature lymphocytes showed positive staining for TdT. EBV hybridization in situ was performed and positive in the LELC (Fig. 5d).

## Discussion

Thymic cysts are uncommon lesions, which are either congenital or acquired in origin. Unilocular cysts are considered to arise from the embryologic remnants, contain clear fluid, have thin walls, and show no evidence of inflammation on histopathologic examination [14, 15]. MTCs are mostly considered to be acquired lesions from inflammatory conditions, and in some cases are associated with thymomas or TCs [6, 9, 15]. The prevalence of MTCs with thymoma or TC are approximately 10.9 and 18.2%, respectively, as reported by Weissferdt and Nakamura [6, 9]. Notably, our 18 cases were obtained from 309 consecutive cases of thymoma and TC, accounting for approximately 5.8%. This relatively lower proportion raises the possibility that thymoma or TC associated with MTC may be more uncommon in the Chinese population. MTCs associated with thymomas or TCs have sporadically been reported in association with type A, type B, micronodular thymoma, SCC, basaloid carcinoma, papillary carcinoma and mucoepidermoid carcinoma [3, 4, 6, 8, 9, 15, 16]. In this study, we add atypical type A thymoma and LELC to MTCs associated with thymomas or TCs which have never been described in the previous reports. Therefore, our data expand the morphologic spectrum of these tumours.

The thymic epithelial tumours feature variable morphologies and clinical behaviours [1]. In the present study, the patients aged 24 to 67 years and had a slight male predominance. In most cases, the patient presented symptoms related to the tumour and its effects on the adjacent organs or related to an inflammatory process. Approximately one third of patients were asymptomatic, and tumours were incidentally detected through radiographic findings. However, only two patients manifested as myasthenia gravis, which indicated that thymomas or TCs developing in the MTCs were likely to be rarely associated with myasthenia gravis. The follow-up data showed that all patients survived at 2 to 79 months after diagnosis (average 26.7 months; median, 17.5 months). Sixteen patients survived without any evidence of recurrence. Given the relatively poor prognosis of conventional thymoma and TC [1], those tumours associated with MTCs seem to show a better clinical behaviours. This is possibly due to the malignant tumour component being relatively limited to the prominent MTC component. Moreover, those tumours with MTCs are accompanied by inflammation, which may allow the tumours to be detected in early stages because of systemic symptoms.

In this study, all 18 tumours were multilocular at histopathologic examination, however, only 14 of 18 lesions were cystic or solid-cystic at gross examination, and only 8 of the 18 lesions appeared unilocular or multilocular on CT or PET-CT scan. The discrepancies among imaging, gross and histopathologic findings suggested that some multiple small cysts may be too small to be revealed on CT and gross examination, and some cysts that were full filled with blood or turbid fluid may present soft-tissue components on CT scan. Thus, imaging and gross examination may have limitations in detecting small MTCs.

Histologically, all cases showed prominent MTC changes, whereas the malignant components were always small-sized and confined. In accordance with WHO histological classification and International Thymic Malignancy Interest Group (ITMIG) consensus, 2 atypical type A thymomas in our study consisted of spindle and oval-shaped tumour cells, showed atypical areas with hypercellularity, enlarged hyperchromatic nuclei, and increased mitotic activity. Though the focal area of the PVS was also seen in one case, the tumour cells showed strong positivity for CD20 which favoured the diagnosis of atypical type A thymoma. The histological diagnosis of other type thymomas and TCs was not controversial, which was further identified by immunohistochemical staining. Simultaneously, LELC was further confirmed by hybridization in situ for EBV detection which was positive. In our series, we added atypical type A thymoma and LELC to MTCs associated with thymomas or TCs which have never been described previously.

The aetiology of MTC development remains unclear. Suster postulated that MTCs arise from the medullary duct epithelium of the cyst which undergoes dilation induced by acquired inflammatory processes [15]. The MTCs of all cases in our series were observed with chronic inflammation, fibrotic changes, haemorrhage and necrosis. Moreover, tumour components were always limited. So we suggest that the formation of MTCs is also closely related to inflammatory conditions rather than mass effects of the tumours. Several previous investigators also supported this proposed aetiology [6, 7, 9]. Furthermore, Nakamura et al. [9] demonstrated that the immunohistochemistry was also in accordance with this hypothesis, the epithelial lining of Hassall corpuscles and MTCs showed similar staining for AE1/AE3, CK13, p63, CK5/6 and D2–40. Our results also confirmed these observations. Since the epithelial cell lining of MTC is considered to be not very different from normal thymic epithelium, any histological type thymoma or TC could arise from the epithelium of the MTC, similar to the thymic epithelial tumour arising from the normal thymic epithelium. Thus, there may be a histogenetic link between MTC and thymic neoplasm. Interestingly, we found that 2 atypical type A and 1 microscopic (type A) thymoma were continuous with the MTC, which suggests that atypical type A and type A thymoma may arise from MTC. However, the continuities were not seen in the other cases. Therefore, it could not be confirmed that there were direct correlations between MTC and thymic neoplasm similar to the thymic epithelial tumour arising from the normal thymic epithelium.

Cystic thymoma is the most important differential diagnosis for thymoma with MTC. The cystic thymoma can be from the cystic dilation of the PVS and absence of epithelial lining within the cystic wall. On the other hand, the MTCs are lined by epithelial cells, but not from degeneration of the PVS, which is the important feature that differentiates cystic thymoma from thymoma with MTC. Second, the differential diagnosis for thymic epithelial tumour associated with MTCs is pure benign MTCs. Appropriate sampling and pathological examination of any cystic lesion plays an important role in the discrimination. For these lesions, total gross sampling should be performed. Lymphomas and germ cell tumours are other types of tumours that often present cystic changes. For these tumours, malignant lymphoid proliferation or germ cells or other characteristic features will be observed through careful sampling and investigation of the cystic structures. In rare instances, metastatic tumours can be associated with MTC, and looking for the primary lesion could be helpful. Additionally, it is to be observed, how to differentiate atypical type A with B3 thymoma. Atypical type A thymoma distinction from spindle cell type B3 thymoma can be difficult. If there is absence of CD20 expression and presence of prominent PVS, which favors the diagnosis of the type B3 thymoma. In contrast, if there is presence of CD20 expression and characteristics of type A thymoma such as presence of focal glands, rosettes or pericytomatous vascular pattern, which favors the diagnosis of atypical type A thymoma. Thus, immunohistochemical staining for CD20 and the characteristic patterns could be helpful for the differential diagnosis between atypical type A and B3 thymoma associated with MTCs.

## Conclusions

Our study suggests that MTCs associated with thymomas or TCs are relatively rare lesions in the anterior mediastinum. The limited malignant tumour component may adhere to adjacent structures of MTC, which was detected unexpectedly. Thus, thorough sampling and morphologic investigation of any cystic lesion of the mediastinum is important to avoid missing a malignant component. Our study adds atypical type A thymoma and LELC to MTC associated with thymoma and TC, which expands the histologic spectrum of thymomas or TCs with MTC. Our study also suggests that thymomas or TCs associated with MTCs have a better prognosis than those tumors without MTCs.

## Abbreviations

CT: Computed tomography
EBV: Epstein-Barr virus
FFPE: Formalin-fixed paraffin-embedded
HE: Haematoxylin and eosin-stained
ITMIG: International Thymic Malignancy Interest Group
LELC: Lymphoepithelioma-like carcinoma
MTC: Multilocular thymic cyst
PVS: Perivascular space
SCC: Squamous cell carcinoma
TC: Thymic carcinoma
WHO: World Health Organization
